# Hematopoietic Stem Cell Transplantation Impact on Patients’ Perceived Quality of Life: A Longitudinal Study

**DOI:** 10.3390/nursrep14010016

**Published:** 2024-01-18

**Authors:** Pablo Ortolá-Alonso, Enric Santacatalina-Roig, Elena Chover-Sierra, Antoni Merelles-Tormo, María Luisa Ballestar-Tarín, Antonio Martínez-Sabater

**Affiliations:** 1 Nursing Department, Facultat d’Infermeria i Podologia, Universitat de València, 46010 Valencia, Spain; paora2@alumni.uv.es (P.O.-A.); enric.santacatalina@uv.es (E.S.-R.); antoni.merelles@uv.es (A.M.-T.); m.luisa.ballestar@uv.es (M.L.B.-T.); antonio.martinez-sabater@uv.es (A.M.-S.); 2 Oncology and Hematology Department, Hospital Clínico Universitario de Valencia, 46010 Valencia, Spain; 3 Nursing Care and Education Research Group (GRIECE), GIUV2019-456, Nursing Department, Universitat de Valencia, 46010 Valencia, Spain; 4 Internal Medicine, Consorcio Hospital General Universitario de Valencia, 46014 Valencia, Spain; 5 Grupo Asociado de Investigación en Cuidados (INCLIVA), Hospital Clínico Universitario de Valencia, 46010 Valencia, Spain

**Keywords:** hematopoietic stem cell transplantation, cancer, oncology, quality of life, nursing

## Abstract

Objective: The aim of this paper was to evaluate the quality of life of adult patients with onco-hematological disease treated with hematopoietic stem cell transplantation up to two years post-transplantation. Method: A quantitative, observational, longitudinal, and analytical study was conducted with 121 participants diagnosed with onco-hematological cancer who underwent hematopoietic stem cell transplantation between October 2017 and September 2019, with a 2-year post-transplantation follow-up, of whom only 39 completed the study. The Functional Assessment of Cancer Therapy—Bone Marrow Transplantation (FACT-BMT) questionnaire and its subscales, Functional Assessment of Cancer Therapy—General (FACT-BMT) and Functional Assessment of Cancer Therapy Trial Outcome Index (FACT-TOI), developed by the Functional Assessment of Chronic Illness Therapy (FACIT) and validated for Spain, were used to assess quality of life. Result: The average age for hematopoietic stem cell transplantation was 54 years, with a majority of male participants. The evaluation of quality of life showed a decrease at the time of hospital discharge, followed by a progressive improvement up to one year after the transplantation. There was a significant difference in the quality of life questionnaire scores between both sexes during all stages of the research, with higher scores in male participants. The length of hospital stay significantly affected patients’ physical and functional well-being, and marital status was related to differences in the perception of quality of life. Conclusions: Despite the initial decrease in quality of life for patients undergoing hematopoietic stem cell transplantation, levels of quality of life similar to baseline are regained one year after the transplantation. Sociodemographic variables are related to how these patients perceive their quality of life. However, further studies with a larger sample size are needed for more precise results.

## 1. Introduction

Hematopoietic stem cell transplantation (HSCT) is a medical intervention to rectify deficiencies or non-viability within a cellular lineage originating from aberrant stem cells. Widely acknowledged as an established curative treatment for various onco-hematological diseases in humans, HSCT is also the preferred therapeutic option for a spectrum of non-oncological conditions, including specific types of anemia, autoimmune diseases, and congenital disorders [1,2].

As reported by the International Agency for Research on Cancer in 2020, global estimates indicated 544,352 cases of non-Hodgkin lymphomas (NHL), 474,519 cases of leukemia, 176,404 cases of multiple myeloma (MM), and 83,087 cases of Hodgkin lymphomas (HL) [3]. Annually, around 50,000 individuals undergo bone marrow transplants [4]. This intricate procedure involves the infusion of hematopoietic cells from a healthy donor, which may be autologous (stem cells derived from the patient’s own body), syngeneic (stem cells from an identical twin), or allogeneic (stem cells from a non-identical donor) [5,6]. Typically, these cells are acquired through the aspiration of bone marrow under general anesthesia in an operating theater. It is noteworthy that alternative sources of hematopoietic stem cells, such as peripheral blood and umbilical cord blood, are also employed in HSCT.

Since its establishment as a therapeutic option in the 1960s and 1970s [6,7], hematopoietic stem cell transplantation (HSCT) has seen an expansion in its indications over the decades, reflecting improvements in efficacy and effectiveness [5,6,8,9,10]. Despite advancements in protocols and treatments that have contributed to a reduction in associated morbidity and mortality [5,11], delivering these therapies in the home setting, which is medically safe and beneficial, has become a trend [12,13].

However, HSCT remains a formidable physical and psychological challenge, exposing individuals to potential risks of adverse reactions that can impact their quality of life (QoL) [14]. Common adverse effects include those associated with aplasia, such as infections, gastrointestinal alterations, pain, asthenia/fragility, allergic reactions, and graft-versus-host disease (GVHD) [6]. Additionally, other complications may arise, including pulmonary toxicity, hepatobiliary toxicity, heart disease, secondary malignant tumors, myelodysplasia/leukemia secondary to treatment, and secondary solid tumors [5,15,16]. Psychological effects, such as fear, anguish, and social isolation, although not necessarily manifesting physically, significantly impact patients socially and emotionally, disrupting their QoL and, at times, resulting in disabling consequences [14,17,18].

Similarly, HSCT exposes patients to a potential decline in their quality of life [19], necessitating a comprehensive assessment of its impact across various dimensions, including its effects on caregivers and families [17,20,21]. Numerous studies have established a correlation between quality of life and clinical complications, underscoring the importance of systematic assessment and ongoing monitoring to inform appropriate decision making [21,22] and the adjustment of care strategies [22,23,24].

Moreover, factors such as hospitalization [25,26] and geographical distance from home [21,25,27,28,29] should be carefully considered, given their potential impact on quality of life [30]. As such, there is a compelling need to broaden the scope of research on this subject, exploring the multifaceted dimensions of the HSCT experience and its implications for patients and their support networks.

The present study aims to evaluate the quality of life of adult patients with onco-hematological disease treated with hematopoietic stem cell transplantation up to two years after the transplantation.

## 2. Materials and Methods

### 2.1. Design

An analytical, observational, longitudinal, and prospective study was developed. Adult patients aged over 18 years were included in the study. For their participation, the requirements were to sign the informed consent form, to suffer from some form of onco-hematological cancer, and to have undergone HSCT at the Hospital Clínico Universitario in Valencia (Spain) from October 2017 to September 2019, taking as a reference the day of transplantation (day 0). No sample selection was performed; all individuals undergoing HCT in 2017 who agreed to participate were included.

This study was conducted under the Declaration of Helsinki and approved by the Ethics Committee of the Hospital Clínico Universitario de Valencia (protocol code 2027/05/25 (377)) on 27 July 2017.

### 2.2. Variables

Data were collected on the sociodemographic characteristics of study participants, including age, sex, marital status, educational level, occupation, average income, cohabitation, family support network, and distance to the hospital; clinical characteristics, such as tumor burden, neutrophil count, diagnosis, type of transplantation, donor–recipient ratio, donor age and sex, source, type of conditioning, chemotherapy treatment, previous transplantations, and comorbidities; and variables related to perceived QoL.

### 2.3. Data Collection Instruments

A self-administered instrument developed by the investigators was used for the sociodemographic and clinical characterization of the patients under study.

To assess the mortality risk associated with transplantation, we employed the Hematopoietic Transplant Comorbidity Index (HCTI), commonly called the “Sorror score”. The HCTI assigns a numerical value between 1 and 3 to each of the comorbidities identified in the patient before transplantation [31].

For the measurement of health-related quality of life (HRQoL), version 4.0 of the Functional Assessment of Cancer Therapy—Bone Marrow Transplantation (FACT-BMT), developed by The Functional Assessment of Chronic Illness Therapy (FACIT), translated and validated for Spain [32] was used. Authorization for its use in this work was granted to the principal investigator.

The FACT-BMT is composed of 50 questions presented in 5 subscales: 4 of them, encompassed by the FACT-G and being able to obtain a total score between 0 and 108, are generic for all cancer patients and group a total of 27 questions related to physical (PWB), social/family (SFWB), functional (FWB), and emotional (EWB) well-being; the remaining 23 questions are related to the subscale dedicated to bone marrow transplantation (BMT), with scores of up to 40 points. Within this tool, the Trial Outcome Index (TOI) was used for the higher quality of information obtained, corresponding to the sum of the scores of the subscales PWB, FWB, and BMT, which can add up to 96 points. Higher test scores indicate better HRQoL [33,34].

To assess in a reduced form the general health status and quality of life of the patients under study, two other numerical scales widely used in oncology were used: the Karnofsky scale (KPS), numbered from 0 to 100, and the ECOG scale, numbered from 0 to 5. In both, the higher the number, the lower the functional impairment to the patient’s life.

### 2.4. Data Analysis

IBM^®^ SPSS^®^ Statistics v25.0 and Microsoft^®^ Excel 2023 were used to analyze the results.

Univariate descriptive analysis of the sociodemographic and clinical characteristics of the subjects was performed using proportions, frequencies, means, ranges, and standard deviations according to the nature of the variables.

The statistical relationship between nominal variables was assessed using the chi-square or Fisher’s exact test.

The Shapiro–Wilk test was used to examine the normality of quantitative variables due to the nature of the sample, and according to its result, the Mann–Whitney U test was needed to determine the statistical relationship between continuous and categorical quantitative variables of 2 categories; the Kruskall–Wallis test was used to determine the statistical relationship between qualitative and quantitative variables.

A linear regression model identified variables statistically influencing perceived QoL results.

A 95% confidence interval was considered for all statistical relationships established, so *p*-values inferior to 0.05 were considered statistically significant.

## 3. Results

Over the two-year study period, 121 patients were recruited and followed up for two years. Data were collected at six stages: at transplantation (day 0), hospital discharge, and at 3-, 6-, 12-, and 24-months post-transplantation.

**Figure 1:**
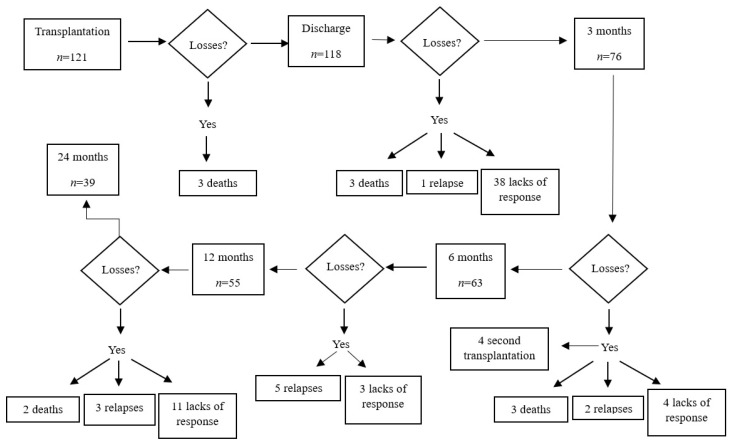
Flow diagram of losses and their justification during the six stages of the study.

As can be seen in Figure 1, after the first study period, at discharge, 118 patients participated; at three months, 76 patients responded to the questionnaires; at six months, 63 patients responded. At 12 months, 55 patients completed the questionnaires, and at 24 months, only 39 patients responded. Losses resulted from 11 deaths, 11 relapses, 4 second transplants, and 56 non-responses. There was a total of 82 withdrawn patients.

### Population Characteristics

Table 1 summarizes the sociodemographic and clinical characteristics of the initial sample (*n* = 121). The mean age of the participants was 54 years, with an age range spanning from 18 to 74 years. The sample comprised 59.5% males and patients were predominantly diagnosed with lymphoma (43%). Approximately 56.67% of the participants underwent autologous transplantation, with the hematopoietic stem cells mainly sourced from peripheral blood (97.38%). Notably, 47.10% of the participants had received prior myeloablative conditioning.

Regarding marital status, the majority (62.8%) identified themselves as married or in a consensual union. Over 76.8% of the participants reported living with at least another person in their household, with an average family support network size of approximately two individuals. Nearly 39.67% resided less than 50 km from the hospital, and 43.8% declared themselves economically active. Among the financially active participants, 46.28% had completed primary education, and the average income for patients in the sample fell within the range of EUR 18,000–22,000 per year. Furthermore, over 71% of the participants had a Karnofsky index of 90 or higher.

Concerning the comparison of the stages of the research which can be seen in Table 2, there is a slight decrease in the mean scores obtained on the scales immediately after the marrow transplantation when the patient was discharged from the hospital. Subsequently, there is evidence of a progressive increase in these scores until they reach figures close to those obtained at admission, one year after transplantation, and then a slight decrease two years after transplantation.

With these data, a two-by-two comparison was performed using the Wilcoxon test for paired samples’ means to analyze the effect of time on perceived QoL, as evidenced by the difference in means for the FACT-BMT scale in the different phases of the study. Here, the differences between the various stages were not statistically significant except for the comparison between admission and discharge, an interval that coincides with the performance of HSCT.

Regarding the statistical analysis of the data, Table 3 shows the result of a linear regression model on the FACT-BMT scale data collected at the time of discharge to identify which variables had a statistically significant effect on the FACT-BMT scale. It shows that the variables “sex” and “marital status” are the only statistically significant variables concerning the differences in the values obtained on the FACT-BMT scale.

The means of the overall perceived QoL measured by the FACT-BMT and those measured by the FACT-TOI and FACT-G scales for the mentioned variables and at the different stages of the research are expressed in Table 4, Table 5 and Table 6.

The results in Table 4 show that in all five post-transplantation stages of the investigation, male patients had significantly higher means on the FACT-BMT rating scale than female patients. The same is true individually for the FACT-TOI and FACT-G subscales, shown in Table 5 and Table 6, respectively, where the means were higher for male patients, with sufficient statistical significance at all stages.

Regarding the variable “marital status”, the regression analysis shown in Table 3 presents it as one of the variables that statistically significantly affected the scores obtained by patients studied on the different scales. However, although it was found that single patients had higher mean scores on the rankings compared to married, separated, and widowed patients when more specific statistical tests were performed for the variables, it was determined that the level of statistical significance was not sufficient to state with adequate certainty that the marital status of the patients affects their perception of QoL.

## 4. Discussion

Hematopoietic stem cell transplantation (HSCT) stands as a complex and aggressive form of treatment involving numerous variables and potential complications that demand meticulous control to safeguard patients’ lives and preserve their quality of life. In the planning phase of this study, the inherent nature of the survey led to the anticipation of foreseeable losses within the initial sample. Ultimately, the study revealed a loss of 82 patients, equating to a reduction of 67.76% from the initial selection. This dropout rate surpassed those identified in studies of a similar nature, such as an observational analytical study conducted in Paraná, Argentina, involving 55 patients undergoing HSCT, which reported a dropout rate of 41.81% [35].

This elevated dropout rate could be attributed to the extended duration of our research, a period during which events such as deaths, relapses, and other conditions potentially leading to exclusion from the study may have occurred. The complex and protracted nature of HSCT treatments may have contributed to these losses, underlining the importance of recognizing and accounting for such factors in future research endeavors.

The assessment of QoL through the FACT-BMT, FACT-G, and FACT-TOI instruments, coupled with the statistical analysis of sociodemographic and clinical variables, provided valuable insights into the impact of hematopoietic stem cell transplantation (HSCT) on the subjects under study. The research outcomes revealed that overall QoL, as measured by the FACT-BMT questionnaire, consistently demonstrated mean scores above 90 points across all stages of the investigation.

Notably, there was an initial decrease in mean scores immediately following HSCT, succeeded by a gradual recovery throughout the treatment process. By one year post-transplantation, the QoL scores surpassed the baseline parameters recorded before transplantation, with a slight reduction observed at the two-year mark. These findings align with prior research, such as studies conducted in Brazil [36] and Spain [30], which evaluated QoL in patients undergoing autologous and allogeneic HSCT. The Spanish study, for instance, reported lower QoL values at two months post-transplantation compared to baseline, followed by an improvement at nine months and a return to baseline values by one year post-transplantation.

Furthermore, this study concludes that the type of HSCT did not significantly impact the quality of life during the examined period, aligning with the results obtained in the present investigation. This consistency in findings across different studies strengthens the understanding of the temporal dynamics of QoL in HSCT recipients. It underscores patients’ resilience in regaining and sometimes surpassing their baseline QoL levels post-transplantation.

Consistent with the observed decline in mean scores on diverse perceived quality of life (QoL) rating scales, a study conducted in the USA in 2023 investigated the trajectory of recovery of QoL and symptom burden up to four years after hematopoietic stem cell transplantation (HSCT) in a cohort of 758 patients. Despite the recovery of baseline FACT-BMT scores one year post-transplantation, the study revealed that many patients continued to report symptoms associated with HSCT, and their QoL remained impaired [37]. This could underscore the complexity of the post-HSCT experience, suggesting that while certain aspects of QoL may rebound, lingering symptoms and challenges persist for many patients beyond the initial recovery period. The findings emphasize the importance of comprehensive, long-term monitoring and support for individuals undergoing HSCT to address persistent symptoms and optimize their overall well-being.

A noteworthy discovery within this study highlights the influence of gender on the perception of quality of life. Although both male and female groups exhibited overall means above 80 on the FACT-BMT scale at all follow-up stages, male patients consistently demonstrated significantly higher scores on all perceived quality of life (QoL) scales (FACT-BMT, FACT-G, and FACT-TOI), particularly in the domains of physical and functional assets. This gender-related disparity aligns with similar findings in both national and international studies. For instance, Ozlem Ovayolu et al., in their research conducted in Pakistan, reported a significant relationship between gender and QoL, with higher scores observed in the male group across various questionnaires [38].

Likewise, a study in Brazil examining QoL during the first year of post-HSCT treatment in 55 patients revealed that male patients reported fewer treatment-associated complications, and their mean scores on QoL questionnaires were generally higher than those of female patients, notably in the physical and functional domains [35]. These consistent findings suggest that gender shapes how patients experience and evaluate their QoL after HSCT. The potential contributions of biological, psychological, or sociocultural factors to these differences underscore the importance of future research endeavors to elucidate this hypothesis. Further exploring the intricate interplay between gender and QoL in HSCT may provide valuable insights for tailored patient care and support strategies.

An additional noteworthy finding from this study underscores the relationship between the length of hospital stay and participants’ perceived quality of life. While this factor did not significantly impact scores on the FACT-BMT scale, it did influence scores on the FACT-TOI subscale. Specifically, as the length of hospital stay increased, patients experienced a decline in physical and functional well-being. This observation resonates with a study conducted in the USA, which similarly concluded that heightened physical and depressive symptoms during hospitalization correlated with a diminished quality of life [39].

These consistent findings emphasize the crucial role of the hospitalization period in shaping the post-HSCT experience, particularly regarding physical and functional aspects of well-being. Recognizing the impact of the length of hospital stay on specific facets of quality of life highlights the importance of tailored interventions and support measures during this critical phase of the transplantation process. Addressing these factors comprehensively may enhance the overall well-being and satisfaction of individuals undergoing HSCT [38,39,40].

This study also delved into the impact of marital status on the perception of quality of life. Initially, single patients seemed to have higher scores on the quality-of-life scales than their married, separated, or widowed counterparts. However, after subjecting the data to more specific statistical tests, insufficient statistical significance was found to confidently conclude that marital status directly influences the perception of quality of life in the context of this study.

These results suggest that while there may be initial differences in perceived quality of life based on marital status, these distinctions may not be robust enough to draw definitive conclusions. The nuanced interplay between marital status and quality of life in the context of HSCT warrants further exploration and consideration of potential confounding factors that may contribute to these observations. Future research endeavors could provide a more comprehensive understanding of how marital status may or may not influence individuals’ post-HSCT quality of life experience.

In consonance with the findings of the current investigation, a study conducted in 2020 involving 15,940 individuals who underwent hematopoietic stem cell transplantation (HSCT) identified a link between participants’ marital status and complications, particularly graft-versus-host disease (GVHD). The occurrence of GVHD was more prevalent in single patients compared to their married and widowed counterparts, ultimately exerting a negative impact on their quality of life [40].

While there is limited literature associating marital status with the perception of quality of life in hematopoietic transplantation, studies in other medical contexts, such as dialysis for chronic renal failure, have explored the relationship between these variables. For instance, a study published in Tarragona [41] reported a connection between marital status and perceived quality of life in dialysis patients. The study revealed poorer results in the domains of vitality, psychological well-being, social function, and pain for individuals who were unmarried or not in a committed relationship. The study suggested that married individuals or those in a committed relationship benefit from emotional support that single or widowed individuals may lack. Additionally, widowed individuals, having experienced the loss of a partner with whom they established solid emotional bonds, tend to exhibit more depressive symptoms and a poorer perceived quality of life.

These findings collectively highlight the potential impact of marital status on both the medical outcomes and the subjective well-being of individuals undergoing various medical treatments, including hematopoietic transplantation. Understanding these relationships can inform supportive care strategies and interventions to enhance patients’ overall experience and outcomes.

Parallel findings from two studies conducted in Chile [42] and Cuba [43], which centered on well-being and perceived social support in older adults, demonstrated that individuals in a committed relationship had higher psychological well-being and greater perceived social support than those without a stable partner. This positive association between relationship status and well-being was linked to a better quality of life.

These studies contribute valuable insights into the broader understanding of the impact of marital status on psychological well-being and social support, which in turn influence the quality of life in distinct populations, including older adults. These findings not only echo similar patterns observed in the context of hematopoietic stem cell transplantation (HSCT) but also underscore the importance of considering the role of social relationships and support systems in shaping individuals’ well-being across various medical and demographic contexts.

Recognizing these consistent trends provides a foundation for future research to delve deeper into the intricate relationship between marital status and quality of life after HSCT. Exploring this connection in more detail can inform targeted interventions and support mechanisms for individuals undergoing HSCT, acknowledging the potential impact of social relationships on their overall well-being.

Indeed, it is crucial to recognize that the pathological process of the disease and the transplantation procedure exert profound effects on family dynamics, influencing various aspects such as functionality and economics [44]. This impact extends beyond the individual patient to encompass the quality of life of their family members [45,46]. The interplay between the disease, the transplantation process, and the broader family context should be a focal point in future research efforts to enhance its depth and quality. By doing so, researchers can gain a more comprehensive understanding of the multifaceted challenges faced by both patients and their families.

Assessing the impact on family dynamics and the quality of life of both patients and their family members is essential for developing integrated and holistic support strategies. These strategies should not only address the medical aspects of the transplantation but also encompass the broader social, economic, and emotional dimensions that influence the overall well-being of individuals and their families. As research advances in this direction, it can contribute to the development of more effective and tailored interventions that better meet the complex needs of patients and their support networks.

Certainly, highlighting the pivotal role of nurses within the multidisciplinary team is crucial. Nurses play a fundamental role in supporting both the patient and their family as they navigate the challenges associated with hematopoietic stem cell transplantation (HSCT). Throughout the various stages of treatment, nurses are instrumental in facilitating adaptation to the new situation, providing holistic care that extends beyond the purely biological aspects of the process.

In the complex landscape of HSCT, nurses serve as integral members of the healthcare team, working to minimize the consequences of stressors associated with the treatment’s intricacies. Their assistance is not limited to addressing the physical aspects of the disease and treatment; instead, they actively intervene in the psychosocial aspects, recognizing the interconnectedness of the patient’s well-being and the challenges faced by their family [45,46,47].

The supportive and comprehensive care provided by nurses is indispensable in promoting the overall well-being of individuals undergoing HSCT and their families. Their expertise, compassion, and dedication contribute significantly to fostering a more positive and manageable experience for patients as they navigate the complexities of the transplantation process.

It is crucial to approach the interpretation of these findings with an awareness of this study’s limitations and the specific context in which it was conducted. One of the primary constraints of the present study was the small number of participants who completed all stages, posing challenges in comparing results with those from larger studies. This limitation may be attributed to the inherent nature of hematopoietic stem cell transplantation (HSCT) as a highly invasive procedure, posing risks to both the physical and psychological well-being not only of the patient but also of the donor [1,6,47,48]. The procedure involves a multitude of complications [8], and its application is not universally suitable for all patients with onco-hematological diseases [5,49].

Furthermore, this study’s geographical context, as evidenced by the data from the National Transplant Organization in Spain, revealed a relatively low number of HSCT cases, particularly in the community of Valencia. This, in turn, affected this study’s sample size, with only 20 HSCTs performed at the Hospital Clínico of Valencia during the study period [50,51,52]. Despite these limitations, it is noteworthy that almost all eligible patients undergoing HSCT during the study period were included, reflecting the challenges associated with the complexity of the procedure and the high dropout rates observed in similar studies.

The small sample size underscores the need for caution in generalizing this study’s findings to broader populations. However, given the intricate nature of HSCT and the challenges associated with participant retention in longitudinal studies of this kind, this study provides valuable insights within the context of its specific limitations. It serves as a foundation for future research efforts aimed at expanding our understanding of the experiences and outcomes associated with HSCT in diverse settings.

Another area to consider is that most of the research about QoL in patients undergoing HSCT, both nationally and internationally, and despite making a clear sociodemographic distinction and categorization of the sample studied, was limited to analyzing differences over time in the questionnaire scores, without establishing relationships with the sociodemographic variables presented. This lack made it challenging to establish relationships of similarity or discrepancy between the results obtained in this study and those of the different studies consulted. This study aims to explain these variables’ roles in the perception of quality of life. It also opens the doors to investigate further how aspects such as gender, marital status, and economic issues influence said perception of life—the quality of life of people undergoing HSCT.

## 5. Conclusions

This research study allowed us to determine which factors can significantly influence the QoL of patients two years after transplantation. A clear positive progression in QoL scores was observed at the different time points after transplantation, with scores above 100 on the FACT-BMT scale and above 70 and 60 on the FACT-G and FACT-TOI scales, respectively.

Among the variables investigated, according to the statistical analysis, sex is the only one that constitutes a differential factor in the perception of quality of life. Other variables could be related; however, further studies would be necessary to determine their influence.

These results demonstrate that despite the physical and emotional challenge of the therapeutic process and the associated risks, patients report a generally satisfactory quality of life in the years following transplantation. These results underline the importance of providing continuous follow-up and support to patients undergoing this treatment, highlighting the need for a comprehensive approach to the care provided, with specialized medical and nursing care and appropriate emotional support, and promoting effective coping strategies.

## Figures and Tables

**Table 1:** Comparison between the first and the last stage of research.

	Hospitalization	24 M
Variables	n = 121 (%)	n = 39 (%)
Sociodemographic		
Age		
18–30 years	12 (9.92%)	4 (10.25%)
31–50 years	28 (23.14%)	10 (25.64%)
51–70 years	74 (61.16%)	23 (58.97%)
More than 70 years	7 (5.78%)	2 (5.13%)
Gender		
Male	72 (59.5%)	27 (69.23%)
Female	49 (40.5%)	12 (30.77%)
Marital status		
Single	27 (22.31%)	9 (23.07%)
Married/Consensual Union	76 (62.8%)	23 (59.97%)
Separated	7 (5.87%)	5 (12.82%)
Widowed	4 (3.30%)	1 (2.56%)
Family network		
One relative	14 (11.57%)	6 (15.38%)
Two relatives	34 (28.1%)	13 (33.33%)
Three or more relatives	49 (40.5%)	17 (43.59%)
Educational level		
Uneducated	2 (1.65%)	
Primary	56 (46.28%)	18 (46.15%)
Secondary	25 (20.66%)	10 (25.64%)
University	24 (19.83%)	8 (20.51%)
Occupation		
Unqualified	47 (38.84%)	9 (23.07%)
Qualified	23 (19%)	14 (35.90%)
Professionals	6 (4.95%)	2 (5.13%)
Civil servant	12 (9.92%)	4 (10.25%)
Self-employed	12 (9.92%)	3 (7.69%)
Student	5 (4.13%)	2 (5.13%)
Economic income (euros/year)		
<18,000	60 (49.59%)	15 (38.46%)
18,000–22,000	7 (5.78%)	2 (5.13%)
22,000–60,000	32 (26.45%)	17 (43.59%)
60,000–90,000	7 (5.78%)	1 (2.56%)
Distance to Hospital (km)		
0–50	48 (39.67%)	18 (46.15%)
50–100	15 (12.39%)	6 (15.38%)
>100	37 (30.58%)	10 (25.64%)
Clinicals		
KPS		
<70	1 (0.82%)	
70	4 (3.30%)	
80	14 (11.57%)	2 (1.65%)
90	86 (71.07%)	33 (27.27%)
100	4 (3.30%)	1 (2.56%)
Sorror score		
0	20 (16.53%)	8 (20.51%)
1	9 (7.44%)	3 (7.69%)
2	25 (20.66%)	10 (25.64%)
3	24 (19.83%)	6 (15.38%)
4	18 (14.87%)	5 (12.82%)
5	7 (5.78%)	2 (5.13%)
6	3 (2.48%)	1 (2.56%)
7	2 (1.65%)	
Diagnosis ^1^		
ALL	3 (2.48%)	1 (2.56%)
AML	19 (15.70%)	5 (12.82%)
CLL	1 (0.82%)	
CML	1 (0.82%)	
MDS	9 (7.42%)	3 (7.69%)
Lymphoma	52 (42.97%)	21 (53.84%)
Multiple myeloma	30 (27.79%)	8 (20.51%)
Amyloidosis	1 (0.82%)	
Myelofibrosis	4 (3.30%)	1 (2.56%)
Embryonal cancer	1 (0.82%)	
Type of HSCT		
Autologous	71 (56.67%)	24 (61.54%)
Allogeneic	29 (23.96%)	10 (25.64%)
Haploidentical	21 (17.35%)	4 (10.25%)
Stem cells’ source		
Bone marrow	1 (0.82%)	
Peripheral Blood	113 (93.38%)	39 (100%)
Conditioning		
Myeloablative	57 (47.10%)	24 (61.54%)
Non-myeloablative	41 (33.88%)	11 (28.20%)

^1^ ALL: Acute lymphoblastic leukemia, AML: Acute myeloblastic leukemia, CLL: Chronic lymphoblastic leukemia, CML: Chronic myeloblastic leukemia, MDS: Myelodysplastic syndrome, and KPS: Karnofsky performance status.

**Table 2:** Results of QoL in the different research stages assessed by Functional Assessment of Cancer Therapy Bone Marrow Transplantation (FACT-BMT), general assessment (FACT-G), and Trial Outcome Index (FACT-TOI).

	Study Stage ^1^					
	Transplantation	Discharge	Three Months	Six Months	12 Months	24 Months
n	121	118	76	63	55	39
FACT-BMT	101.12	97.34	97.76	100.21	104.54	97.19
FACT-G	76.20	73.44	73.16	74.36	77.68	71.68
FACT-TOI	62.81	58.08	60.42	62.81	66.68	61.58

^1^ The values shown are the arithmetic means of the scores obtained by the total sample at each stage; FACT-BMT: assessment of the FACT scale specific to bone marrow transplant; FACTG: general assessment (physical well-being/social and family well-being/emotional well-being/functional well-being); FACT-TOI: assessment of physical and functional well-being.

**Table 3:** Coefficients of the multiple linear model explaining the changes in quality of life perceived by patients at the time of hospital discharge (stage 2).

Variable ^1^	β	SD	Sig.
Age	0.271	0.281	*p* = 0.339
Sex	−20.147	6.603	<0.01
Marital status	−8.789	4.305	<0.015
Cohabitation	9.452	8.509	*p* = 0.272
Level of education	−0.303	4.435	*p* = 0.946
Occupation	1.749	1.845	*p* = 0.348
Income	−4.065	3.748	*p* = 0.284
Diagnosis	0.199	1.281	*p* = 0.877
Previous transplants	4.600	6.680	*p* = 0.494
Family network	−3.133	3.284	*p* = 0.345
Type of transplantation	2.388	3.835	*p* = 0.536
Marrow source	0.782	21.232	*p* = 0.971

^1^ Dependent variable: FACT-BMT at discharge; β: coefficient of change; SD: standard deviation.

**Table 4:** Differences in the results obtained on FACT-BMT in each of the stages of the study depending on gender and marital status.

	Functional Assessment of Cancer Therapy—Bone Marrow Transplantation (FACT-BMT)					
		Discharge	3 M	6 M	12 M	24 M
N		118	76	63	55	39
Gender						
Male	Average	102.77	105.50	107.57	111.83	103.47
	SD ^1^	15.157	19.925	20.628	20.052	23.868
Female	Average	89.43	86.76	89.02	91.055	84.02
	SD	21.7	20.611	19.744	22.199	16.138
*p*-value		<0.001	<0.001	<0.01	<0.001	<0.05
Marital Status						
Single	Average	101.08	100.91	100.86	107.56	108.31
	SD	22.094	22.140	22.115	19.377	26.010
Married/consensual union	Average	98.06	97.18	101.90	107.66	98.43
	SD	18.167	23.077	23.093	20.297	24.042
Separated	Average	88.56	88.04	90.23	88.10	82.10
	SD	5.978	18.671	11.726	24.089	11.749
Widowed	Average	89.66	103.91	90.16	85	74.05
	SD	28.479	22.745	30.641		
*p*-value		0.214	0.601	0.591	0.186	0.262

^1^ Mann–Whitney U test; SD: standard deviation.

**Table 5:** Differences in the results obtained on FACT-TOI in each of the stages of the study depending on gender and marital status.

	Functional Assessment of Cancer Therapy—Trial Outcome Index (FACT-TOI)					
		Discharge	3 M	6 M	12 M	24 M
N		118	76	63	55	39
Gender ^a^						
Male	Average	62.05	66.53	68.30	72.01	66.88
	SD	11.993	15.212	15.081	13.576	16.873
Female	Average	52.29	51.74	54.46	56.82	50.44
	SD	16.265	15.192	14.597	14.429	12.628
*p*-value		<0.001	<0.001	<0.01	<0.001	<0.05
Marital Status ^b^						
Single	Average	61	63.58	65.33	70.49	72.03
	SD	17.074	14.731	13.096	12.788	17.704
Married/consensual union	Average	58.26	59.36	63.85	68.66	61.97
	SD	14.019	18.086	18.065	14.428	17.780
Separated	Average	53.49	55.28	53	51.8	48.40
	SD	6.508	15.304	7.071	17.398	5.594
Widowed	Average	52.5	69.5	51	52	46.25
	SD	21.625	16.263	18.384		
*p*-value		0.454	0.514	0.286	0.075	0.133

^a^ Mann–Whitney U test; ^b^ Kruskall–Wallis test; SD: standard deviation.

**Table 6:** Differences in the results obtained on FACT-G in each of the stages of the study depending on gender and marital status.

	Functional Assessment of Cancer Therapy—General (FACT-G)					
		Discharge	3 M	6 M	12 M	24 M
N		118	76	63	55	39
Gender ^a^						
Male	Average	77.26	78.26	78.76	82.49	76.20
	SD	11.186	14.382	16.706	15.022	17.987
Female	Average	67.88	65.93	67.68	68.78	62.18
	SD	16.286	15.037	14.713	15.377	12.217
*p*-value		<0.001	<0.01	<0.01	<0.01	<0.05
Marital Status ^b^						
Single	Average	76.04	75.38	75.53	79.06	77.77
	SD	16.085	16.382	16.686	13.348	20.640
Married/consensual union	Average	74.21	72.95	74.89	79.94	73.07
	SD	13.556	16.119	17.496	15.202	17.844
Separated	Average	66.21	65.18	69.23	66.5	62.10
	SD	4.368	13.432	11.983	18.580	11.227
Widowed	Average	67.41	77.41	70.66	67	52.8
	SD	18.125	17.795	22.863		
*p*-value		0.136	0.474	0.805	0.358	0.320

^a^ Mann–Whitney U test; ^b^ Kruskall–Wallis test; SD: standard deviation.

## Data Availability

Data are available upon request from the corresponding author.

## Public Involvement Statement

There was no public involvement in any aspect of this research.

## Guidelines and Standards Statement

This manuscript was drafted in accordance with the STROBE guidelines for observational research.

## References

[1] Balassa K., Danby R., Rocha V. (2019). Haematopoietic stem cell transplants: Principles and indications. Br. J. Hosp. Med..

[2] Godino C., Scotti A., Marengo A., Battini I., Brambilla P., Stucchi S., Slavich M., Salerno A., Fragasso G., Margonato A. (2022). Effectiveness and cost-efficacy of diuretics home administration via peripherally inserted central venous catheter in patients with end-stage heart failure. Int. J. Cardiol..

[3] World Health Organization International Agency for Research on Cancer (IARC), Globocan 2012. https://www.iarc.fr/.

[4] Von Ah D., Spath M., Nielsen A., Fife B. (2016). The Caregiver’s Role Across the Bone Marrow Transplantation Trajectory. Cancer Nurs..

[5] Rifón J.J. (2006). Transplant of hemopoietic progenitors. An. Sist. Sanit. Navar..

[6] Ascensión B. (2009). Pilar Moreno Psychosocial risks and psychological intervention in the transplanted bone marrow patients. Psicooncologia.

[7] Ochoa-Fernández B., Galán-Gómez V., Mestre C., González-Vicent M., Pascual A., Alonso L., Regueiro A., Plaza M., Hurtado J.M.P., Benito A. (2022). Haploidentical hematopoietic stem cell transplantation in pediatric and adolescent patients: A study of the Spanish hematopoietic stem cell transplantation group (GETH). Bone Marrow Transplant..

[8] Jiménez-Ubieto A., Grande C., Caballero D., Yáñez L., Novelli S., Hernández-Garcia M.T., Manzanares M., Arranz R., Ferreiro J.J., Bobillo S. (2019). Autologous stem cell transplantation may be curative for patients with follicular lymphoma with early therapy failure without the need for immunotherapy. Hematol. Stem Cell Ther..

[9] Murthy G.S.G., Mehta P., Jethava Y., Dhakal I., Makhoul I. (2014). Trends in hospitalization outcomes of elderly patients undergoing allogeneic stem cell transplantation for acute myeloid leukemia/myelodysplastic syndrome (AML/MDS). J. Clin. Oncol..

[10] Torrent A., Ferrá C., Batlle M., Hidalgo F., Jiménez-Lorenzo M.-J., Ribera J.-M. (2020). Prospective follow-up of adult long-term survivors of allogeneic haematopoietic stem cell transplantation. Med. Clín..

[11] Peters W.P., Ross M., Vredenburgh J.J., Meisenberg B., Marks L.B., Winer E., Kurtzberg J., Bast R.C., Jones R., Shpall E. (1993). High-dose chemotherapy and autologous bone marrow support as consolidation after standard-dose adjuvant therapy for high-risk primary breast cancer. J. Clin. Oncol..

[12] Bergkvist K., Fossum B., Johansson U.-B., Mattsson J., Larsen J. (2017). Patients’ experiences of different care settings and a new life situation after allogeneic haematopoietic stem cell transplantation. Eur. J. Cancer Care.

[13] Kharfan-Dabaja M.A., Roy V., Murthy H., Fischer D., Mohty R., Greathouse A., Brown A., Moreno K., Godsey E., Higginbotham J.M. (2023). Post autologous hematopoietic cell transplant care in the “home sweet home” setting: A treatment paradigm shift. Hematol. Stem Cell Ther..

[14] Barba P., Elorza I. (2019). Allogeneic stem cell transplantation in the era of novel therapies for acute lymphoblastic leukaemia. Med. Clín..

[15] Detrait M., De Berranger E., Dulery R., Ménard A.-L., Thépot S., Toprak S.K., Turlure P., Yakoub-Agha I., Guillaume T. (2020). Complications hépatobiliaires dans le contexte de l’allogreffe de cellules hématopoïétiques: Recommandations de la Société francophone de greffe de moelle et de thérapie cellulaire (SFGM-TC). Bull. Cancer.

[16] Zhang X., Wang J., Liu Y., Liu J., Wang B., Zhang Q., Guan W., Zhang H., Xu L., Liu G. (2022). Long-term survivors demonstrate superior quality of life after haploidentical stem cell transplantation to matched sibling donor transplantation. J. Transl. Med..

[17] Janicsák H., Ungvari G.S., Gazdag G. (2021). Psychosocial aspects of hematopoietic stem cell transplantation. World J. Transplant..

[18] Buchbinder D., Khera N. (2021). Psychosocial and financial issues after hematopoietic cell transplantation. Hematology.

[19] Mathanda R.R., Hamilton B.K., Rybicki L., Advani A.S., Colver A., Dabney J., Ferraro C., Hanna R., Kalaycio M., Lawrence C. (2020). Quality-of-Life Trajectories in Adolescent and Young Adult versus Older Adult Allogeneic Hematopoietic Cell Transplantation Recipients. Biol. Blood Marrow Transplant..

[20] Rocha V.d., Proença S.d.F.F.S., Marques A.d.C.B., Pontes L., Mantovani M.d.F., Kalinke L.P. (2016). Social impairment of patients undergoing hematopoietic stem cell transplant. Rev. Bras. Enferm..

[21] Young L. (2013). The family experience following bone marrow or blood cell transplantation. J. Adv. Nurs..

[22] Bryant A.L., Drier S.W., Lee S., Bennett A.V. (2018). A systematic review of patient reported outcomes in phase II or III clinical trials of myelodysplastic syndromes and acute myeloid leukemia. Leuk. Res..

[23] Jim H.S.L., Quinn G.P., Barata A., Cases M., Cessna J., Gonzalez B., Gonzalez L., Koskan A., Montiel-Ishino F., Pidala J. (2014). Caregivers’ quality of life after blood and marrow transplantation: A qualitative study. Bone Marrow Transplant..

[24] Gifford G., Sim J., Horne A., Ma D. (2013). Health status, late effects and long-term survivorship of allogeneic bone marrow transplantation: A retrospective study. Intern. Med. J..

[25] Fernández-Avilés F., Carreras E., Urbano-Ispizua A., Rovira M., Martínez C., Gaya A., Granell M., Ramiro L., Gallego C., Hernando A. (2006). Case-Control Comparison of At-Home to Total Hospital Care for Autologous Stem-Cell Transplantation for Hematologic Malignancies. J. Clin. Oncol..

[26] Dunavin N., Mau L.-W., Meyer C.L., Divine C., Abdallah A.-O., Leppke S., D’Souza A., Denzen E., Saber W., Burns L.J. (2020). Health Care Reimbursement, Service Utilization, and Outcomes among Medicare Beneficiaries with Multiple Myeloma Receiving Autologous Hematopoietic Cell Transplantation in Inpatient and Outpatient Settings. Biol. Blood Marrow Transplant..

[27] Martino M., Ciavarella S., De Summa S., Russo L., Meliambro N., Imbalzano L., Gallo G.A., Moscato T., Messina G., Ferreri A. (2018). A Comparative Assessment of Quality of Life in Patients with Multiple Myeloma Undergoing Autologous Stem Cell Transplantation Through an Outpatient and Inpatient Model. Biol. Blood Marrow Transplant..

[28] Murthy G.S.G., Hari P.N., Szabo A., Pasquini M., Narra R., Khan M., Abedin S., Chhabra S., Dhakal B., D’Souza A. (2018). Outcomes of Reduced-Intensity Conditioning Allogeneic Hematopoietic Cell Transplantation Performed in the Inpatient versus Outpatient Setting. Biol. Blood Marrow Transplant..

[29] Gutiérrez-García G., Rovira M., Arab N., Gallego C., Sánchez J., Álvarez M., Ayora P., Domenech A., Borràs N., Rodríguez-Lobato L.G. (2020). A reproducible and safe at-home allogeneic haematopoietic cell transplant program: First experience in Central and Southern Europe. Bone Marrow Transplant..

[30] Seixas M.R., Rodríguez L.L., Fernández J.M.P., Moncada M.V., Quijano-Campos J.C. (2014). Quality of life related to health in patients with hematopoietic stem cell transplantation. Index Enferm..

[31] Sorror M.L., Maris M.B., Storb R., Baron F., Sandmaier B.M., Maloney D.G., Storer B. (2005). Hematopoietic cell transplantation (HCT)-specific comorbidity index: A new tool for risk assessment before allogeneic HCT. Blood.

[32] Cella D., Hernandez L., Bonomi A.E., Corona M., Vaquero M., Shiomoto G., Baez L. (1998). Spanish Language Translation and Initial Validation of the Functional Assessment of Cancer Therapy Quality-of-Life Instrument. Med. Care.

[33] Webster K., Cella D., Yost K. (2003). The Functional Assessment of Chronic Illness Therapy (FACIT) Measurement System: Properties, applications, and interpretation. Health Qual. Life Outcomes.

[34] Montan I., Löwe B., Cella D., Mehnert A., Hinz A. (2018). General Population Norms for the Functional Assessment of Chronic Illness Therapy (FACIT)-Fatigue Scale. Value Health.

[35] Marques A.d.C.B., Szczepanik A.P., Machado C.A.M., Santos P.N.D., Guimarães P.R.B., Kalinke L.P. (2018). Hematopoietic stem cell transplantation and quality of life during the first year of treatment. Rev. Latino-Am. Enferm..

[36] dos Santos C.L.T., Sawada N.O., dos Santos J.L.F. (2011). Evaluation of the health-related quality of life of hematopoietic stem cell transplantation patients. Rev. Latino-Am. Enferm..

[37] D’Souza A., Brazauskas R., Stadtmauer E.A., Pasquini M.C., Hari P., Bashey A., Callander N., Devine S., Efebera Y., Ganguly S. (2022). Trajectories of quality of life recovery and symptom burden after autologous hematopoietic cell transplantation in multiple myeloma. Am. J. Hematol..

[38] Ovayolu O., Ovayolu N., Kaplan E., Pehlivan M., Karadağ G. (2013). Symptoms and Quality of Life: Before and after stem cell transplantation in cancer. Pak. J. Med Sci..

[39] Kenzik K., Huang I.-C., Rizzo J.D., Shenkman E., Wingard J. (2014). Relationships among symptoms, psychosocial factors, and health-related quality of life in hematopoietic stem cell transplant survivors. Support. Care Cancer.

[40] Tay J., Beattie S., Bredeson C., Brazauskas R., He N., Ahmed I.A., Aljurf M., Askar M., Atsuta Y., Badawy S. (2020). Pre-Transplant Marital Status and Hematopoietic Cell Transplantation Outcomes. Curr. Oncol..

[41] Gomà A.S., Peris P.A., Alcario A.B.R. (2010). Calidad de vida en pacientes con insuficiencia renal crónica en tratamiento con diálisis. Rev. Soc. Esp. Enferm. Nefrol..

[42] Vivaldi F., Barra E. (2012). Psychological Well-Being, Perceived Social Support and Health Perception Among Older Adults. Ter. Psicol..

[43] Alfonso Figueroa L., Soto Carballo D., Santos Fernández N.A. (2016). Calidad de vida y apoyo social percibido en adultos mayores. Rev. Cienc. Méd..

[44] Rodrigue J.R., Hoffmann R.G., Macnaughton K., Graham-Pole J., Andres J.M., A Novak D., Fennell R.S. (1996). Mothers of children evaluated for transplantation: Stress, coping resources, and perceptions of family functioning. Clin. Transplant..

[45] Wochna V. (1997). Anxiety, needs, and coping in family members of the bone marrow transplant patient. Cancer Nurs..

[46] Matsubara T.C., de Carvalho E.C., Canini S.R.M.d.S., Sawada N.O. (2007). Family crisis in the context of bone marrow transplantation: An integrative review. Rev. Latino-Am. Enferm..

[47] Nakamura Z.M., Nash R.P., Quillen L.J., Richardson D.R., McCall R.C., Park E.M. (2019). Psychiatric Care in Hematopoietic Stem Cell Transplantation. Psychosomatics.

[48] El-Jawahri A., Chen Y., Brazauskas R., He N., Lee S.J., Knight J.M., Majhail N., Buchbinder D., Schears R.M., Wirk B.M. (2017). Impact of pre-transplant depression on outcomes of allogeneic and autologous hematopoietic stem cell transplantation. Cancer.

[49] Sancho J.M., Ribera J.M., Oriol A., Batlle M., Flores A., Rodríguez L., Torrabadella M., Millá F., Feliu E. (2003). Reasons for not carrying out stem cell transplantation in patients referred to a transplant unit. Med. Clín..

[50] Fundación Josep Carreras Memoria Anual REDMO Fundación Carreras. Fundación Josep Carreras Contra la Leucemia 2023. https://www.fcarreras.org/es/MemoriaREDMO_2022.

[51] Organización Nacional de Trasplantes (2023). Balance de Actividad de Donación y Trasplante en España. https://www.ont.es/2023/02/10/balanceont20230117-3/.

[52] Garcés-Carrasco A.M., Santacatalina-Roig E., Carretero-Márquez C., Chover-Sierra E., Martínez-Sabater A., Balaguer-López E. (2023). Post-Transplant Complications in Patients Undergoing Autologous Hematopoietic Cell Transplantation (HCT)—A Comparative Analysis of Home Care versus Hospitalized Patients. Medicina.

